# Molecular, biofilm and motility characterization of *Acinetobacter baumannii* isolated from a neonatal intensive care unit at a children’s hospital in South China

**DOI:** 10.3389/fcimb.2025.1705282

**Published:** 2025-11-28

**Authors:** Zelin Yu, Hongbo Fu, Yanqiong Zhou, Xiajiong Luo, Yuebin Wu, Zejian Kuang, Danwei Cai, Xiuming Wu, Xiaobing Hong

**Affiliations:** 1 Department of Pharmacy, The Second Affiliated Hospital of Shantou University Medical College, Shantou, China; 2 Department of Critical Care Medicine, The Second Affiliated Hospital of Shantou University Medical College, Shantou, China

**Keywords:** *Acinetobacter baumannii*, biofilm, motility, multidrug resistance, children

## Abstract

**Background:**

Multidrug-resistant *Acinetobacter baumannii* (MDR-AB) poses a critical threat in neonatal intensive care units (NICUs), however few studies have reported the molecular characterization of *A. baumannii* isolates from the NICU in China. This study aims to describe the molecular, biofilm and motility characteristics related to carbapenem resistance genes in *A. baumannii* isolates from the NICU.

**Methods:**

Following an outbreak in March–April 2024, 21 A*. baumannii* isolates from neonatal and environmental sources were collected. These isolates underwent antimicrobial susceptibility testing, whole-genome sequencing (WGS) for resistance gene profiling and multi-locus sequence typing (MLST). Biofilm formation and twitching motility were measured in representative strains exposed to subinhibitory concentrations of gentamicin, levofloxacin, and meropenem.

**Results:**

All 21 A*. baumannii* isolates were carbapenem-resistant *Acinetobacter baumannii* (CRAB), showing resistance to cephalosporins, aminoglycosides, and quinolones. WGS identified 25 resistance genes, including the universal *bla*_OXA-23_, *bla*_OXA-66_, *bla_ADC-25_*and *bla*_TEM-1D_, aminoglycoside resistance genes (*armA*, *aph genes*), macrolide resistance genes (*mphE*, *msrE*), and tetracycline (*tetB*) resistance genes. Efflux pumps (*adeABC*, *adeFGH*, *adeIJK*) were ubiquitous. Two sequence types emerged: ST195 (20 isolates) and ST1959 (1 isolate), with high genetic similarity suggesting nosocomial transmission. ST1959 exhibited stronger biofilm formation than ST195. Biofilm formation of ST1959 was significantly enhanced by gentamicin but inhibited by levofloxacin. Meropenem suppressed only the ST195 biofilms. Twitching motility was markedly reduced in ST1959 and also decreased after levofloxacin exposure.

**Conclusion:**

This NICU outbreak was driven by CRAB ST195 harboring a conserved resistome. Strain-specific differences in biofilm formation and motility under antibiotic stress highlight the interplay between genetic lineage and phenotypic adaptability. Levofloxacin demonstrates dual anti-biofilm and anti-motility effects, which could guide targeted infection control strategies against high-risk *A. baumannii* clones.

## 1 Introduction

*Acinetobacter baumannii* (AB) is a Gram-negative, non-fermenting bacterium that has been frequently identified in intensive care units(ICUs) in recent years. It predominantly infects critically ill, immunocompromised patients, those undergoing invasive procedures, and individuals receiving broad-spectrum antibiotics. AB is associated with a range of diseases, including meningitis, pneumonia, peritonitis, and bacteremia (Muller et al., 2023). The mortality rate of invasive AB infections remains alarmingly high. Recent data from systematic reviews and meta-analyses indicate that crude mortality rates for carbapenem-resistant A. baumannii (CRAB) infections can range from 30% to 44%, particularly among vulnerable populations such as neonates and immunocompromised individuals (Lee et al., 2017; Tacconelli et al., 2018; Luo et al., 2025). CRAB has shown an alarming upward trend in global epidemiology over the past decade, with significant regional variations. High endemicity is reported in Asia, Europe, and the Americas (Mohd Sazlly Lim et al., 2019; Luo et al., 2024). Notably, China faces a critical challenge with CRAB, where reported resistance rates to carbapenems frequently exceed 60-70% in some regions, establishing it as a priority pathogen for infection control (Wang et al., 2024). A similar concerning trend is observed in neighboring countries, including India, Thailand where the high prevalence of CRAB contributes to substantial public health burdens (Kamolvit et al., 2015; Kumar et al., 2018).

The success of *A. baumannii* as a hospital-acquired pathogen is largely attributable to its remarkable ability to develop multidrug resistance and persist in the environment. Key virulence factors include biofilm formation, which protects bacterial communities from antibiotics and host immune responses, and surface-associated motility, which facilitates colonization and spread (Jain et al., 2019; Armalyte et al., 2023). These mechanisms confer resistance to desiccation, nutritional stress, and disinfection protocols, thereby contributing to the persistence of endemic clones and sustained hospital outbreaks.

Although frequently reported as a major pathogen in adults, *A. baumannii* is equally menacing in neonatal intensive care unit (NICU) (Vogiantzi et al., 2024). Risk factors for NICU-acquired AB infections include low birth weight, prolonged NICU stay, umbilical catheterization, central venous catheterization, assisted ventilation, and prior antibiotic exposure (Wei et al., 2015; Phatigomet et al., 2022). Several outbreaks of CRAB in the NICU have been documented in the last decade (Zarrilli et al., 2012; Tsiatsiou et al., 2015; Wei et al., 2015; Ulu-Kilic et al., 2018).

Therapeutic options for CRAB infections remain severely limited, complicating clinical management. The emergence of extensively drug-resistant (XDR) strains has led to the reliance on last-resort agents, such as colistin and tigecycline, often used in combination therapy (Isler et al., 2019). Although such combinations have demonstrated some clinical success, their efficacy is often offset by concerns regarding toxicity (e.g., nephrotoxicity from colistin) and the emergence of resistance. Recent advances with novel beta-lactam/beta-lactamase inhibitor combinations (e.g., cefiderocol, sulbactam-durlobactam) offer promising alternatives, demonstrating enhanced *in vitro* activity and improved clinical outcomes in certain settings. However, their accessibility and high cost limit widespread use, especially in resource-limited settings (Iovleva et al., 2025). This underscores that infection control and prevention remain the cornerstone of managing CRAB.

Enhancing awareness and establishing robust epidemiological surveillance, including molecular characterization of circulating strains, are fundamental to successful infection control and are particularly imperative in high-risk environments like the NICU. However, despite the significant burden of CRAB, detailed studies on the molecular epidemiology, biofilm formation, and motility characteristics of CRAB isolates specifically from NICUs in South China remain scarce. The present study aims to address this gap by providing a comprehensive molecular, biofilm, and motility characterization of CRAB clinical isolates obtained from a children’s hospital NICU in South China. We report the distribution of carbapenem resistance genes and examine the correlation between resistance profiles and virulence traits, thereby offering valuable insights to inform local infection control strategies and optimize antimicrobial therapy.

## 2 Materials and methods

### 2.1 Bacterial strains and culture conditions

A total of twenty-one clinical isolates of *A. baumannii* investigated in this study were collected during the period of 2024 by medical laboratories of tertiary care hospitals in Shantou city, Guangdong province, China. All isolates were cultured in Luria-Bertani (LB) broth with shaking at 37°C. Antimicrobial susceptibility testing was performed using Mueller-Hinton (MH) broth or agar plates.

### 2.2 Antimicrobial susceptibility testing

Antimicrobial susceptibility testing was performed using the BD Phoenix-100 Automated Microbiology System (BD Diagnostics, Sparks, MD, USA) with the NMIC/ID-402 panel for Gram-negative bacteria, following the manufacturer’s instructions. Results were interpreted according to the Clinical and Laboratory Standards Institute guidelines (CLSI M100, 2024). The tested antibiotics included those pre-configured in the panel, such as imipenem (10 µg), meropenem (10 µg), amikacin (30 µg), ampicillin-sulbactam (10/10 µg); piperacillin-tazobactam (36 µg); ceftazidime (30 µg); cefotaxime (30 µg); cefepime (30 µg); Aztreonam; CIP: ciprofloxacin (5µg); AMK: amikacin (30 µg); gentamicin (10 µg); Levofloxacin (5 µg); SXT: trimethoprim-sulfamethoxazole (1.25/23.75 µg); colistin (30 µg).

### 2.3 Whole genome sequencing, genome annotation analysis and visualization

Whole-genome sequencing was performed using the Illumina HiSeq 2500 second-generation high-throughput sequencing platform with a 2×100 bp paired-end configuration. The Illumina-based workflow comprised three primary steps: library preparation from extracted nucleic acids, amplification sequencing, and quality control. Raw sequencing reads in FASTQ format were assembled into contigs (FASTA files) using SPAdes v2.0 software on a Linux/CentOS system. Command-line execution was performed according to the SPAdes (v3.16.1) documentation (https://github.com/ablab/spades).

ResFinder (version 4.6.0) (https://cge.food.dtu.dk/services/ResFinder/) from the Center for Genomic Epidemiology (CGE, DTU) was employed to identify antimicrobial resistance genes in the contigs of 21 CRAB strains. Finally, a heatmap visualizing the distribution of resistance genes across the 21 CRAB strains was generated using MORPHEUS (https://software.broadinstitute.org/morpheus/).

### 2.4 Multi-locus sequence typing

Multi-locus sequence typing (MLST) was performed in silico on the assembled genomes using the MLST software (v2.25.0, https://github.com/tseemann/mlst) on a Linux system. The software automatically queried the PubMLST database (https://pubmlst.org/organisms/acinetobacter-baumannii) against the Oxford scheme. The seven housekeeping genes (*gltA*, *gyrB*, *gdhB*, *recA*, *cpn60*, *gpi*, and *rpoD*) were aligned and compared using BLAST algorithms. A unique integer identifier was assigned to each allele of these genes. The combination of these integers (allelic profile) defined the Sequence Type (ST) for each isolate. Whole-genome single nucleotide polymorphism (SNP) analysis was performed using CSI Phylogeny 1.4 from the Center for Genomic Epidemiology (CGE; https://cge.food.dtu.dk/services/CSIPhylogeny/). The complete, high-quality genome of Acinetobacter baumannii ATCC 19606 (GenBank accession: CP045113.1) and ATCC 17978 (GenBank accession: CP053094.1) was used as the reference for the whole-genome alignment. This standard reference strain was selected to ensure consistent and reproducible SNP calling, facilitating reliable phylogenetic inference and cross-study comparisons. A maximum-likelihood phylogenetic tree was constructed from the core genome SNP alignment using FigTree v1.4.4. The genetic relatedness among the 21 A. baumannii strains was assessed based on the resulting tree topology to evaluate their homology and elucidate potential transmission pathways.

### 2.5 Biofilm formation assays

Biofilm production was evaluated using the crystal violet (CV) staining assay as previously described by O’Toole and Kolter with slight modifications (O'Toole and Kolter, 1998). Briefly, A. baumannii overnight cultures were adjusted to a turbidity equivalent to 0.5 McFarland standard in 0.85% saline solution. For the quantitative assessment of biofilm formation, biofilms were formed in 24-well flat-bottom polystyrene plates. Bacterial suspensions were added to the wells and were statically incubated at 37°C for 48 h. After incubation, the planktonic cells were removed, and the adhered biofilms were gently washed twice with phosphate-buffered saline (PBS), air-dried, and stained with 1 mL of 0.7% (w/v) crystal violet solution per well for 15 min. The excess stain was rinsed off with distilled water, and the bound CV was solubilized with 1 mL of 33% (v/v) glacial acetic acid per well. The optical density (OD) of the solubilized CV was then measured at 600 nm using a Tecan Infinite M200 Pro microplate reader. Additionally, for qualitative visualization and representative imaging of the biofilm structures, the assay was performed in parallel using sterile 15 mL round-bottom polystyrene tubes. The processing, staining, and solubilization steps of the biofilm were identical to those used in the 24-well plates.

The OD values from the 24-well plate assay were corrected for background staining. This was done by subtracting the value for CV bound to uninoculated Müller Hinton Broth (MHB) control wells*. E. coli* J53 and *P. aeruginosa* PAO1 were included as the negative and positive controls, respectively, in both setups. Furthermore, all experiments were performed in triplicate and repeated independently on three different days with consistent results.

### 2.6 Surface-associated motility

The motility assay was conducted using Motility Test Medium, which was inoculated on the surface and incubated overnight at 37°C. This procedure followed the manufacturer’s detailed instructions to ensure accurate and reliable results. Modified LB broth (tryptone-10 g/l; NaCl -5 g/l; yeast extract-5 g/l) with either 0.4 or 0.8% agar was used for all the motility assays. Freshly grown cultures were stabbed to enable spread of bacteria on the surface of the medium (0.4% semisolid) for swarming motility and the interphase between the bottom of the Petri dish and medium (0.8% semisolid) for twitching motility, as described previously (Clemmer et al., 2011). Plates were prepared on the same day as the inoculation. After inoculation, the plates were sealed with parafilm and incubated at 37°C for 48 h.

## 3 Results

### 3.1 Clinical information of the neonates

The primary case of neonatal in the NICU was identified on 23rd March, following which up to 15 April 2024, sudden surge of 8 neonates (Total 21 isolates) due to *A. baumannii* were noticed from NICU (**Table 1**). A total of 21 strains were identified, comprising of 9 female, 8 male, and 3 of unknown gender. The youngest neonate was 2 hours old and the oldest was 22 days old. The isolates were collected from various sources: nose swabs (6 isolates), bed sheets (4 isolates), ventilator patient ends (2 isolates), endotracheal tube, ETT (4 isolates), hands (3 isolates), condensate water (1 isolate), and sputum (1 isolate).

**Table 1:** Clinical characteristics of the neonates with carbapenem-resistant *Acinetobacter baumannii* infection.

Patient	Sample no.	Sex	Age	Administrative office	Sampling type	type	ST	MLST profiles	Sample date
P1	GD03286	F	22 days	NICU	Sputum	Infection	195	1-3-3-2-2-96-3	2024-3-23
P2	GD03287	M	22 H	NICU	Tracheal catheter	Infection	195	1-3-3-2-2-96-3	2024-3-23
P3	GD03288	F	10 H	NICU	Tracheal catheter	Infection	1959	1-3-3-2-2-335-3	2024-3-23
P3	GD03289	F	10 hours	NICU	Nose swab	Infection	195	1-3-3-2-2-96-3	2024-3-23
P2	GD03290	M	2 hours	NICU	Nose swab	Infection	195	1-3-3-2-2-96-3	2024-3-23
P1	GD03291	F	22 days	NICU	Nose swab	Infection	195	1-3-3-2-2-96-3	2024-3-23
P4	GD03283	F	14 days	NICU	Bed sheet	Environmental	195	1-3-3-2-2-96-3	2024-3-23
P1	GD03292	F	22 days	NICU	Bed sheet	Environmental	195	1-3-3-2-2-96-3	2024-3-23
P3	GD03285	F	10 hours	NICU	Hand	Colonization	195	1-3-3-2-2-96-3	2024-3-23
P4	GD03293	F	14 days	NICU	Hand	Colonization	195	1-3-3-2-2-96-3	2024-3-24
P5	GD03294	F	7 days	NICU	Tracheal catheter	Infection	195	1-3-3-2-2-96-3	2024-4-2
P6	GD03295	NR	NR	NICU	Nose swab	Infection	195	1-3-3-2-2-96-3	2024-4-11
P6	GD03296	NR	NR	NICU	Hand	Colonization	195	1-3-3-2-2-96-3	2024-4-11
P6	GD03297	NR	NR	NICU	Bed sheet	Environmental	195	1-3-3-2-2-96-3	2024-4-11
P7	GD03298	M	NR	NICU	Nose swab	Infection	195	1-3-3-2-2-96-3	2024-4-11
P7	GD03299	M	NR	NICU	Bed sheet	Environmental	195	1-3-3-2-2-96-3	2024-4-11
P7	GD03300	M	NR	NICU	Ventilator (patient)	Infection	195	1-3-3-2-2-96-3	2024-4-11
P8	GD03301	M	NR	NICU	Nose swab	Infection	195	1-3-3-2-2-96-3	2024-4-15
P8	GD03302	M	NR	NICU	Ventilator (patient)	Infection	195	1-3-3-2-2-96-3	2024-4-15
P8	GD03303	M	NR	NICU	Condensed water	Environmental	195	1-3-3-2-2-96-3	2024-4-15
P7	GD03284	M	NR	NICU	Tracheal catheter	Infection	195	1-3-3-2-2-96-3	2024-4-9

# NR, not recorded; F, femal; M, man.

### 3.2 Antimicrobial susceptibility testing, MIC determination

All 21 A*. baumannii* isolates collected in this study were confirmed as CRAB strains based on their resistance to imipenem and meropenem. These isolates exhibited multidrug resistance, as summarized in **Table 2**, with consistent resistance to piperacillin, piperacillin-tazobactam, ceftazidime, cefepime, and gentamicin. Additionally, they showed varying levels of sensitivity (MIC) to sulfamethoxazole and polymyxin.

**Table 2:** Antimicrobial susceptibility profiles of the 21 *Acinetobacter baumannii* isolates causing infection at the neonatal intensive care unit.

Sample No.	MIC((μg/mL)												
	SAM	TZP	CTX	FEP	ATM	IPM	MEM	AMK	GEN	CIP	LV	SXT	COL
GD03286	>16(R)	>64(R)	>32(R)	>16(R)	>16(R)	>8(R)	>8(R)	>32(R)	>8(R)	>2(R)	>8(R)	≤0.5(S)	1
GD03287	>16(R)	>64(R)	>32(R)	>16(R)	>16(R)	>8(R)	>8(R)	>32(R)	>8(R)	>2(R)	>8(R)	≤0.5(S)	1
GD03288	>16(R)	>64(R)	>32(R)	>16(R)	>16(R)	>8(R)	>8(R)	>32(R)	>8(R)	2(R)	>8(R)	≤0.5(S)	≤0.5
GD03289	>16(R)	>64(R)	>32(R)	>16(R)	>16(R)	>8(R)	>8(R)	>32(R)	>8(R)	2(R)	8(R)	≤0.5(S)	≤0.5
GD03290	>16(R)	>64(R)	>32(R)	>16(R)	>16(R)	>8(R)	>8(R)	>32(R)	>8(R)	>2(R)	>8(R)	≤0.5(S)	1
GD03291	>16(R)	>64(R)	>32(R)	>16(R)	>16(R)	>8(R)	>8(R)	>32(R)	>8(R)	>2(R)	>8(R)	≤0.5(S)	1
GD03283	>16(R)	>64(R)	>32(R)	>16(R)	>16(R)	>8(R)	>8(R)	>32(R)	>8(R)	>2(R)	>8(R)	≤0.5(S)	1
GD03292	>16(R)	>64(R)	>32(R)	>16(R)	>16(R)	>8(R)	>8(R)	>32(R)	>8(R)	>2(R)	>8(R)	≤0.5(S)	1
GD03285	>16(R)	>64(R)	>32(R)	>16(R)	>16(R)	>8(R)	>8(R)	>32(R)	>8(R)	>2(R)	>8(R)	≤0.5(S)	≤0.5
GD03293	>16(R)	>64(R)	>32(R)	>16(R)	>16(R)	>8(R)	>8(R)	>32(R)	>8(R)	2(R)	>8(R)	≤0.5(S)	≤0.5
GD03294	>16(R)	>64(R)	>32(R)	>16(R)	>16(R)	>8(R)	>8(R)	>32(R)	>8(R)	>2(R)	>8(R)	≤0.5(S)	≤0.5
GD03295	>16(R)	>64(R)	>32(R)	>16(R)	>16(R)	>8(R)	>8(R)	>32(R)	>8(R)	>2(R)	>8(R)	≤0.5(S)	1
GD03296	>16(R)	>64(R)	>32(R)	>16(R)	>16(R)	>8(R)	>8(R)	>32(R)	>8(R)	>2(R)	>8(R)	≤0.5(S)	1
GD03297	>16(R)	>64(R)	>32(R)	>16(R)	>16(R)	>8(R)	>8(R)	>32(R)	>8(R)	>2(R)	>8(R)	1(S)	1
GD03298	>16(R)	>64(R)	>32(R)	>16(R)	>16(R)	>8(R)	>8(R)	>32(R)	>8(R)	2(R)	>8(R)	≤0.5(S)	≤0.5
GD03299	>16(R)	>64(R)	>32(R)	>16(R)	>16(R)	>8(R)	>8(R)	>32(R)	>8(R)	>2(R)	>8(R)	≤0.5(S)	1
GD03300	>16(R)	>64(R)	>32(R)	>16(R)	>16(R)	>8(R)	>8(R)	>32(R)	>8(R)	>2(R)	>8(R)	≤0.5(S)	≤0.5
GD03301	>16(R)	>64(R)	>32(R)	>16(R)	>16(R)	>8(R)	>8(R)	>32(R)	>8(R)	>2(R)	8(R)	≤0.5(S)	≤0.5
GD03302	>16(R)	>64(R)	>32(R)	>16(R)	>16(R)	>8(R)	>8(R)	>32(R)	>8(R)	>2(R)	>8(R)	≤0.5(S)	1
GD03303	>16(R)	>64(R)	>32(R)	>16(R)	>16(R)	>8(R)	>8(R)	>32(R)	>8(R)	2(R)	8(R)	≤0.5(S)	≤0.5
GD03284	>16(R)	>64(R)	>32(R)	>16(R)	>16(R)	>8(R)	>8(R)	>32(R)	>8(R)	2(R)	>8(R)	≤0.5(S)	≤0.5

# MIC was determined by the BD Phoenix-100 system. Antimicrobial susceptibility results in parentheses were based on MIC interpretive standards of the CLSI (2024) for *Acinetobacter* spp.: R, resistant; I, intermediate; S, susceptible; SAM, ampicillin-sulbactam; TZP, piperacillin-tazobactam; CAZ, ceftazidime; CTX, cefotaxime; FEP, cefepime; ATM, Aztreonam; IPM, imipenem; MEM, meropenem; CIP, ciprofloxacin; AMK, amikacin; GEN, gentamicin; LV, Levofloxacin; SXT, trimethoprim-sulfamethoxazole; COL, colistin (polymyxin E).

### 3.3 The distribution of drug-resistant genotypes carried by *Acinetobacter baumannii*

Analysis of 21 A*. baumannii* via WGS identified 25 resistance genes, comprising carbapenem resistance gene, β-Lactamase, multiple efflux pump-related, and antibiotic modified enzyme resistance genes (**Table 3**). All 21 strains (100.0%) had the same four β-lactam resistant genes: *bla*_ADC-25_, *bla*_OXA-23_,*bla*_OXA-66_, and *bla*_TEM-1D._ Among these, the acquired carbapenemase gene *bla*_OXA-23_ and the intrinsic *bla*_OXA-66_ are primarily responsible for conferring carbapenem resistance. The *bla*_ADC-25_ (AmpC) and *bla*_TEM-1D_ genes contribute to the broader-spectrum resistance to other β-lactams and cephalosporins. Among the six aminoglycoside resistance genes all (100.0%) carried *armA*, *aph (3”) Ib*, *aph (3 ‘) - Ia*, and *aph (6) - Id*. 100% of the macrolide resistance genes *mph (E)* and *msr (E)* were detected, as well as 100% of the *gyrAS81L* and *parCS84L* Quinolone Resistance-Determining Region (*QRDR*) mutations. Additionally, 21 strains carried the tetracycline resistance gene *tet (B)*. Efflux pump-related genes, such as *adeABC*, *adeFGH*, *adeIJK* and *acrAB*. No sulfonamide resistance gene (*sul1*), trimethoprim resistance gene (*dfrA1*, *dfrA7*, *dfrA19*), chloramphenicol resistance gene (*cat A1*, *catB8*, *cmlA1*), Rifampicin resistance gene (*ARR-2*), or Colistin resistance gene (*mcr-1/2/3*) were found.

**Table 3:** The distribution of drug-resistant genotypes carried by *Acinetobacter baumannii*.

Res Genes	GD03286	GD03287	GD03288	GD03289	GD03290	GD03291	GD03283	GD03292	GD03293	GD03294	GD03295	GD03296	GD03297	GD03298	GD03299	GD03300	GD03301	GD03302	GD03303	GD03284
*blaADC-25*	**1**	**1**	**1**	**1**	**1**	**1**	**1**	**1**	**1**	**1**	**1**	**1**	**1**	**1**	**1**	**1**	**1**	**1**	**1**	**1**
*blaGES-11*	0	0	0	0	0	0	0	0	0	0	0	0	0	0	0	0	0	0	0	0
*blaNDM-1*	0	0	0	0	0	0	0	0	0	0	0	0	0	0	0	0	0	0	0	0
*blaOXA-23*	**1**	**1**	**1**	**1**	**1**	**1**	**1**	**1**	**1**	**1**	**1**	**1**	**1**	**1**	**1**	**1**	**1**	**1**	**1**	**1**
*blaOXA-58*	0	0	0	0	0	0	0	0	0	0	0	0	0	0	0	0	0	0	0	0
*blaOXA-66*	**1**	**1**	**1**	**1**	**1**	**1**	**1**	**1**	**1**	**1**	**1**	**1**	**1**	**1**	**1**	**1**	**1**	**1**	**1**	**1**
*blaTEM-1D*	**1**	**1**	**1**	**1**	**1**	**1**	**1**	**1**	**1**	**1**	**1**	**1**	**1**	**1**	**1**	**1**	**1**	**1**	**1**	**1**
*blaCARB-2*	0	0	0	0	0	0	0	0	0	0	0	0	0	0	0	0	0	0	0	0
*blaOXA-301*	0	0	0	0	0	0	0	0	0	0	0	0	0	0	0	0	0	0	0	0
*ant(2’’)-Ia*	0	0	0	0	0	0	0	0	0	0	0	0	0	0	0	0	0	0	0	0
*aph(3’)-Ia*	**1**	**1**	**1**	**1**	**1**	**1**	**1**	**1**	**1**	**1**	**1**	**1**	**1**	**1**	**1**	**1**	**1**	**1**	**1**	**1**
*aph(3’)-VIa*	0	0	0	0	0	0	0	0	0	0	0	0	0	0	0	0	0	0	0	0
*aph(3’’)-Ib*	**1**	**1**	**1**	**1**	**1**	**1**	**1**	**1**	**1**	**1**	**1**	**1**	**1**	**1**	**1**	**1**	**1**	**1**	**1**	**1**
*aph(6)-Id*	**1**	**1**	**1**	**1**	**1**	**1**	**1**	**1**	**1**	**1**	**1**	**1**	**1**	**1**	**1**	**1**	**1**	**1**	**1**	**1**
*armA*	**1**	**1**	**1**	**1**	**1**	**1**	**1**	**1**	**1**	**1**	**1**	**1**	**1**	**1**	**1**	**1**	**1**	**1**	**1**	**1**
*tetA*	0	0	0	0	0	0	0	0	0	0	0	0	0	0	0	0	0	0	0	0
*tetB*	**1**	**1**	**1**	**1**	**1**	**1**	**1**	**1**	**1**	**1**	**1**	**1**	**1**	**1**	**1**	**1**	**1**	**1**	**1**	**1**
*tetG*	0	0	0	0	0	0	0	0	0	0	0	0	0	0	0	0	0	0	0	0
*tetX*	0	0	0	0	0	0	0	0	0	0	0	0	0	0	0	0	0	0	0	0
*tet(39)*	0	0	0	0	0	0	0	0	0	0	0	0	0	0	0	0	0	0	0	0
*aac(6’)-Ib-cr*	0	0	0	0	0	0	0	0	0	0	0	0	0	0	0	0	0	0	0	0
*gyrAS81L*	**1**	**1**	**1**	**1**	**1**	**1**	**1**	**1**	**1**	**1**	**1**	**1**	**1**	**1**	**1**	**1**	**1**	**1**	**1**	**1**
*parCS84L*	**1**	**1**	**1**	**1**	**1**	**1**	**1**	**1**	**1**	**1**	**1**	**1**	**1**	**1**	**1**	**1**	**1**	**1**	**1**	**1**
*catA1*	0	0	0	0	0	0	0	0	0	0	0	0	0	0	0	0	0	0	0	0
*catB8*	0	0	0	0	0	0	0	0	0	0	0	0	0	0	0	0	0	0	0	0
*cmlA1*	0	0	0	0	0	0	0	0	0	0	0	0	0	0	0	0	0	0	0	0
*sul1*	0	0	0	0	0	0	0	0	0	0	0	0	0	0	0	0	0	0	0	0
*sul2*	0	0	0	0	0	0	0	0	0	0	0	0	0	0	0	0	0	0	0	0
*mphE*	**1**	**1**	**1**	**1**	**1**	**1**	**1**	**1**	**1**	**1**	**1**	**1**	**1**	**1**	**1**	**1**	**1**	**1**	**1**	**1**
*msrE*	**1**	**1**	**1**	**1**	**1**	**1**	**1**	**1**	**1**	**1**	**1**	**1**	**1**	**1**	**1**	**1**	**1**	**1**	**1**	**1**
*ARR-2*	0	0	0	0	0	0	0	0	0	0	0	0	0	0	0	0	0	0	0	0
*dfrA1*	0	0	0	0	0	0	0	0	0	0	0	0	0	0	0	0	0	0	0	0
*dfrA7*	0	0	0	0	0	0	0	0	0	0	0	0	0	0	0	0	0	0	0	0
*dfrA10*	0	0	0	0	0	0	0	0	0	0	0	0	0	0	0	0	0	0	0	0
*mcr-1/2/3*	0	0	0	0	0	0	0	0	0	0	0	0	0	0	0	0	0	0	0	0
*adeABC*	**1**	**1**	**1**	**1**	**1**	**1**	**1**	**1**	**1**	**1**	**1**	**1**	**1**	**1**	**1**	**1**	**1**	**1**	**1**	**1**
*adeFGH*	**1**	**1**	**1**	**1**	**1**	**1**	**1**	**1**	**1**	**1**	**1**	**1**	**1**	**1**	**1**	**1**	**1**	**1**	**1**	**1**
*adeIJK*	**1**	**1**	**1**	**1**	**1**	**1**	**1**	**1**	**1**	**1**	**1**	**1**	**1**	**1**	**1**	**1**	**1**	**1**	**1**	**1**
*acrAB*	**1**	**1**	**1**	**1**	**1**	**1**	**1**	**1**	**1**	**1**	**1**	**1**	**1**	**1**	**1**	**1**	**1**	**1**	**1**	**1**
*craA*	**1**	**1**	**1**	**1**	**1**	**1**	**1**	**1**	**1**	**1**	**1**	**1**	**1**	**1**	**1**	**1**	**1**	**1**	**1**	**1**
*smvA*(*amvA*)	**1**	**1**	**1**	**1**	**1**	**1**	**1**	**1**	**1**	**1**	**1**	**1**	**1**	**1**	**1**	**1**	**1**	**1**	**1**	**1**
*emrAB*	**1**	**1**	**1**	**1**	**1**	**1**	**1**	**1**	**1**	**1**	**1**	**1**	**1**	**1**	**1**	**1**	**1**	**1**	**1**	**1**
*tetA*	0	0	0	0	0	0	0	0	0	0	0	0	0	0	0	0	0	0	0	0
*tetB*	**1**	**1**	**1**	**1**	**1**	**1**	**1**	**1**	**1**	**1**	**1**	**1**	**1**	**1**	**1**	**1**	**1**	**1**	**1**	**1**
*tetG*	0	0	0	0	0	0	0	0	0	0	0	0	0	0	0	0	0	0	0	0
*tetX*	0	0	0	0	0	0	0	0	0	0	0	0	0	0	0	0	0	0	0	0
*tetX1*	0	0	0	0	0	0	0	0	0	0	0	0	0	0	0	0	0	0	0	0
*abeM*	**1**	**1**	**1**	**1**	**1**	**1**	**1**	**1**	**1**	**1**	**1**	**1**	**1**	**1**	**1**	**1**	**1**	**1**	**1**	**1**
*abeS*(*emrE*)	**1**	**1**	**1**	**1**	**1**	**1**	**1**	**1**	**1**	**1**	**1**	**1**	**1**	**1**	**1**	**1**	**1**	**1**	**1**	**1**
*msrE*	**1**	**1**	**1**	**1**	**1**	**1**	**1**	**1**	**1**	**1**	**1**	**1**	**1**	**1**	**1**	**1**	**1**	**1**	**1**	**1**
*macAB*	**1**	**1**	**1**	**1**	**1**	**1**	**1**	**1**	**1**	**1**	**1**	**1**	**1**	**1**	**1**	**1**	**1**	**1**	**1**	**1**

Bold values are used to visually distinguish between different types of drug resistance genes and have no specific statistical or quantitative significance.

### 3.4 Phylogenetic tree diagram of 21 strains of *Acinetobacter baumannii*

**Figure 1:**
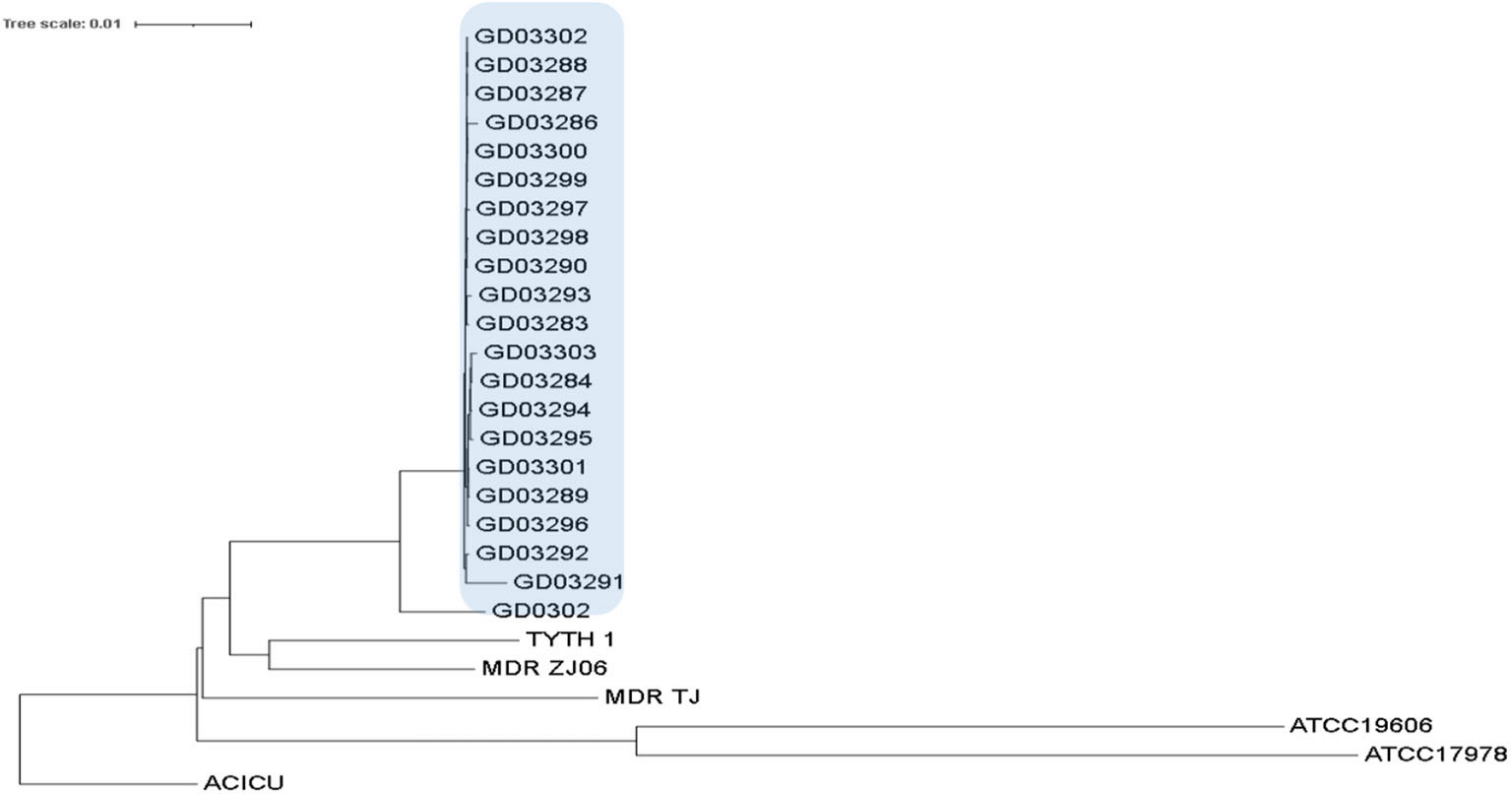
Phylogenetic tree diagram of 20 Ab isolates (without Ab_GD03285).

The distribution of the isolates has been clearly depicted in **Figure 1**. Notably, two distinct sequence types (STs) were identified: ST195, which includes GD03286 and several other isolates, and ST1959, which consists only of GD03288. Despite the classification into different STs, the strains exhibited remarkable similarities, with the only significant variation being found in the *gpi* gene sequence. This close genetic relationship raises concerns regarding the potential for nosocomial transmission, suggesting these strains may have been circulated within healthcare settings, thereby posing a risk for infection among patients.

### 3.5 Biofilm of the strain GD03286 and GD03288 in the presence or absence of antibiotics

**Figure 2:**
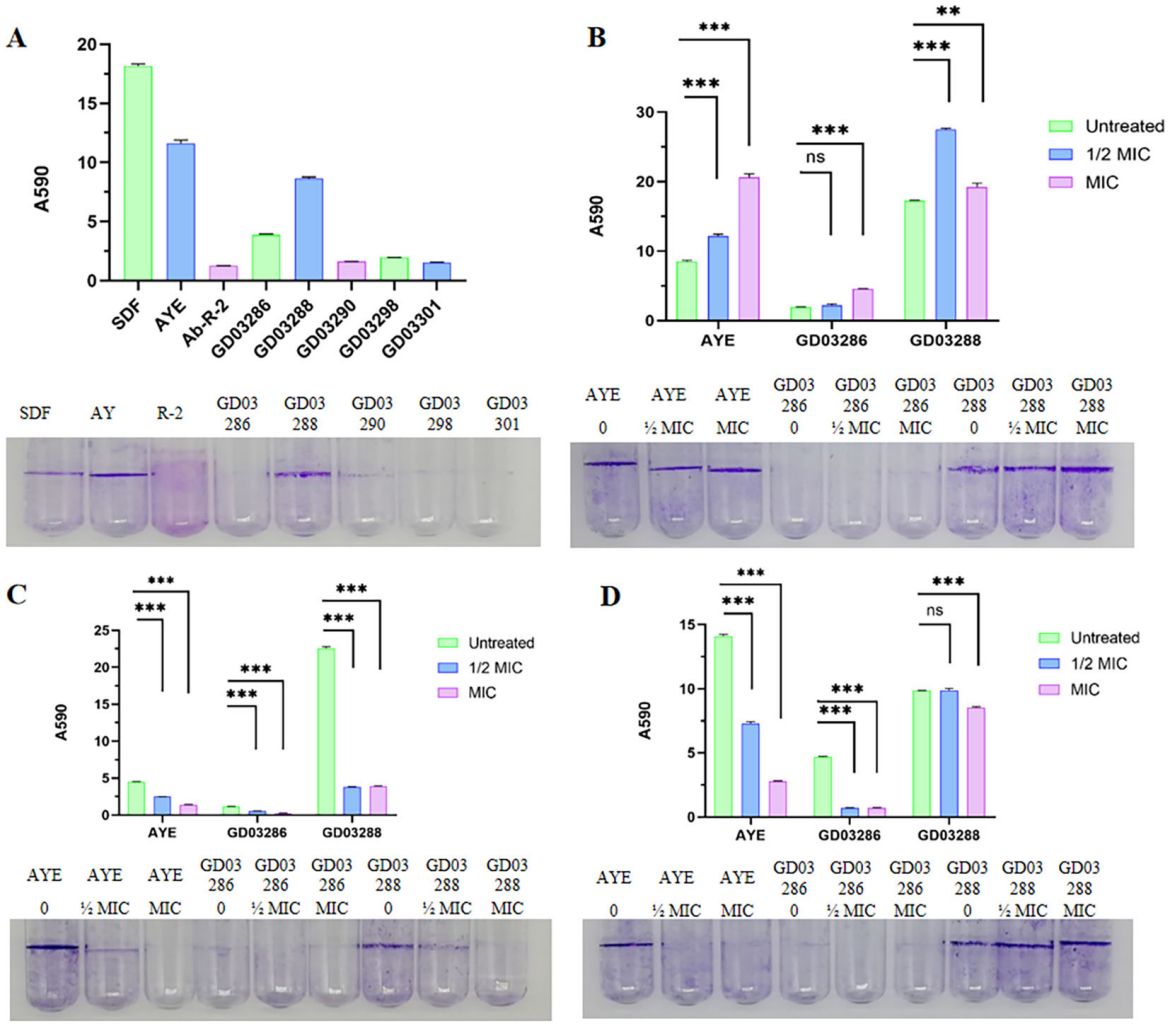
Biofilm of the strain in the presence or absence of antibiotics. **(A)** OD=1 1% inoculant, 37°C stand still for 48h, no antibiotic; **(B)** OD=1 1% inoculant, 37°C stand still for 48h, with Gentamicin, ½xMIC=4 μg/mL and MIC = 8 μg/mL; **(C)** OD = 1 1% inoculant, 37°C stand still for 48h, with Levofloxacin, ½xMIC=4 μg/mL and MIC = 8 μg/mL; **(D)** OD=1 1% inoculant, 37°C stand still for 48h, with Meropenem, ½xMIC=0.5 μg/mL and MIC = 1 μg/mL. ns: not significant; "**“:P<0.05, "***":P<0.01.

Representative polystyrene tubes displayed a range of biofilm formations, which were subsequently stained with crystal violet following a 24-hour incubation period to visualize the extent of biofilm development. Notably, when compared to the control strain AYE, strain GD03288, which belongs to sequence type ST1959 and was isolated from an endotracheal tube (ETT), exhibited a markedly enhanced capacity for biofilm formation. This was in stark contrast to strain GD03286, classified as ST195 and isolated from sputum samples, as well as other clinical isolates, which demonstrated a comparatively weaker biofilm formation ability, as shown in **Figure 2A**.

Furthermore, the presence of gentamicin at concentrations of both ½xMIC and MIC significantly augmented the biofilm formation capacity of strain GD03288, as illustrated in **Figure 2B**. Conversely, levofloxacin, administered at the same concentrations, was shown to have a pronounced inhibitory effect on biofilm formation, as depicted in **Figure 2C**. Additionally, meropenem, at concentrations of ½xMIC and MIC, effectively inhibited the biofilm formation of strain GD03286; however, it did not exert a significant impact on the biofilm development of strain GD03288 at the ½xMIC concentration, as shown in **Figure 2D**. These findings underscore the complex interactions between antibiotic treatments and biofilm formation in these bacterial strains, highlighting the potential for strain-specific responses to antibiotic exposure.

### 3.6 Motility of the strain GD03286 and GD03288 in the presence or absence of antibiotics

**Figure 3:**
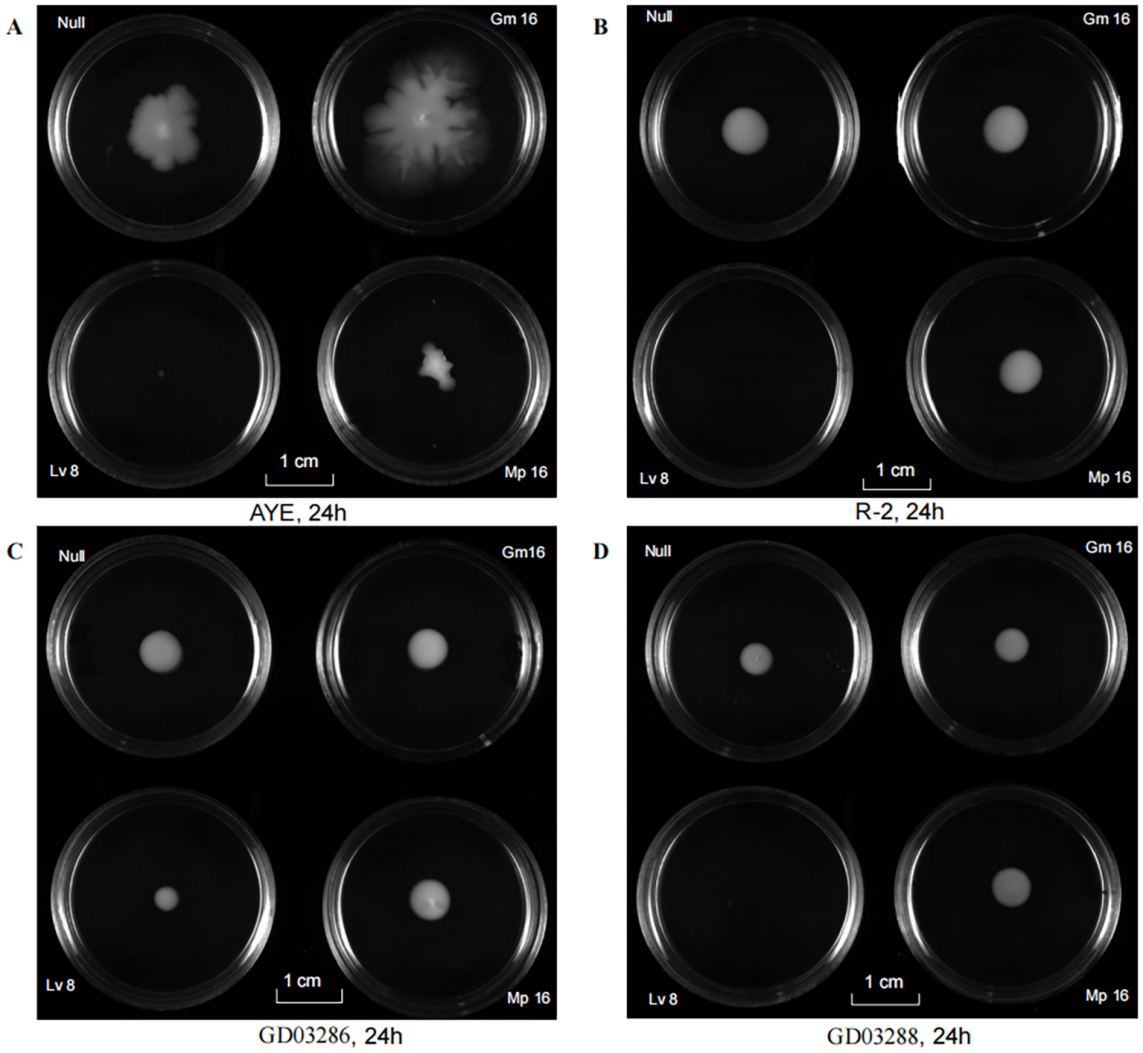
Motility of the strain in the presence or absence of antibiotics. **(A)** Motility suppression effect against representative strains (AYE) of Gm, Lv and Mp. **(B)** Motility suppression effect against representative strains (R-2) of Gm, Lv and Mp. **(C)** Motility suppression effect against representative strains (GD03286) of Gm, Lv and Mp. **(D)** Motility suppression effect against representative strains (GD03288) of Gm, Lv and Mp. Gm, Gentamicin; Lv, Levofloxacin; Mp, Meropenem.

The twitching motility of *A. baumannii* was assessed qualitatively by visually evaluating the characteristic subsurface hazy growth that developed around the inoculation site, providing a clear indication of the bacterial movement. Notably, the antibiotic levofloxacin(Lv 8 µg/mL) was observed to substantially inhibit this motility pattern (**Figure 3**), highlighting its potential as a therapeutic agent. Furthermore, the clinical isolate GD03288 (**Figure 3C**) exhibited a visibly weaker and more constrained motility phenotype compared to reference strains AYE (**Figure 3A**) and R-2 (**Figure 3B**), as well as the more motile clinical strain GD03286. This reduced motility in GD03288 may indicate an adaptation or resistance mechanism distinct from other strains.

## 4 Discussion

This study provides a comprehensive analysis of *A. baumannii* isolates from a 2024 outbreak in a neonatal intensive care unit (NICU) in South China. The extensive multidrug-resistant (MDR) phenotype observed, which included resistance to carbapenems, cephalosporins, aminoglycosides, and quinolones, was largely explained by the complex resistome uncovered through whole-genome sequencing (WGS).

Resistance to CRAB is complex and involves the upregulation of efflux pumps, reduced expression of membrane porin proteins, alteration of drug targets, and production of carbapenemases (Pannek et al., 2006; Poirel and Nordmann, 2006; Lee et al., 2017). All the *A. baumannii* isolates in this study were found to harbor carbapenemase resistance genes *bla*_OXA-23_ and *bla*_OXA-66_, with a detection rate of 100%. *bla*_OXA-23_ and *bla*_OXA-66_ are both Ambler D-class enzymes, and transmission of *bla*_OXA-23_ is usually through plasmids or chromosomes (Mugnier et al., 2010; Ayoub Moubareck and Hammoudi Halat, 2020). *ISAba1*, an insertion sequence located upstream of *bla*_OXA-23_, suggested to have a coordinating effect on it (Royer et al., 2018). *bla*_OXA-23_ and *bla*_OXA-66_ may be key contributors to CRAB’s resistance to carbapenem antibiotics in this hospital, which is in line with reports from both domestic and international sources (Sung et al., 2016). Recent studies have revealed a high prevalence of the *bla*_OXA-23_ gene in *Acinetobacter* spp., and it is hypothesized that the plasmids *pAZJ221* and *Tn2009* are likely to be involved in its horizontal transmission (Liu et al., 2015). Supporting this, Hu et al. (2011) reported that all 17 isolates from a NICU outbreak carried the *bla*_OXA-23_ gene, underscoring the value of detecting this gene for managing nosocomial infections in neonatology (Hu et al., 2011).

This multidrug-resistant phenotype was further compounded by the universal presence of *bla_ADC-25_* (an inherent, often overexpressed AmpC cephalosporinase) and *bla_TEM-1D_* (an extended-spectrum β-lactamase), which collectively contribute to high-level resistance to β-lactams. Resistance to aminoglycosides was primarily mediated by the 16S rRNA methyltransferase gene *armA*, which confers high-level resistance to virtually all aminoglycosides, correlating perfectly with our susceptibility testing results. The concomitant presence of *aph* genes may further augment this phenotype (Rizk and Abou El-Khier, 2019; Ababneh et al., 2023). Furthermore, the detection of macrolide (*mphE*, *msrE*) and tetracycline (*tetB*) resistance genes explains the resistance to these drug classes. Critically, the ubiquitous presence of three efflux pump systems (*adeABC*, *adeFGH*, *adeIJK*) constitutes a fundamental pillar of resistance. *AdeABC*, in particular, is a key contributor to resistance against multiple drug classes (including aminoglycosides, fluoroquinolones, and tetracyclines), and its overexpression is closely linked to the challenges in developing efflux pump inhibitors for clinical use (Sharma et al., 2023). The superposition of these multifactorial resistance mechanisms endows this clone with formidable adaptability, posing a severe challenge to clinical management in the NICU.

Molecular typing identified the outbreak was dominated by the internationally disseminated high-risk clone ST195 (20/21), with one isolate belonging to ST1959. ST195, a member of the global clonal complex CC92/CC2, is a predominant strain responsible for recent hospital outbreaks in China, particularly in NICUs (Percival et al., 2015). The high genetic similarity strongly indicates clonal nosocomial transmission, underscoring the urgent need for enhanced infection control measures in this vulnerable clinical setting.

Beyond antimicrobial resistance, bacterial persistence and dissemination capacity are critical virulence determinants, therefore, we investigated two phenotypes critically associated with pathogenicity and environmental adaptability: biofilm formation and twitching motility. A particularly intriguing and potentially clinically significant observation was that isolates recovered from the inner surface of neonatal endotracheal tubes exhibited the strongest biofilm formation and the weakest motility. This finding is highly congruent with existing theory. Biofilms are protective communities formed by bacteria on the surface of medical devices (e.g., ETTs, central venous catheters) and are central to the persistence of device-associated infections, making them notoriously difficult to eradicate (Percival et al., 2015; Arciola et al., 2018; Deshmukh-Reeves et al., 2025). In the specific niche of an ETT, robust biofilm formation is advantageous for resisting antibiotics and host immune defenses, enabling long-term colonization and recurrent infection. Conversely, reduced motility may signify a switch from a “dissemination mode” to a “colonization mode” whereby bacteria reallocate energy resources from the synthesis of motility organelles (e.g., pili) to the production of biofilm matrix and adherence. Our results suggest that within the actual clinical environment, strains residing at critical sites of infection may be selected for this high-biofilm, low-motility phenotype.

Notably, despite being part of the same outbreak, the ST1959 isolate demonstrated significantly stronger biofilm formation than the predominant ST195 clone, indicating that even within a highly clonal population, minor genotypic variations can lead to significant phenotypic divergence, potentially influencing niche adaptation (Penesyan et al., 2021).

Exposure to subinhibitory concentrations of antibiotics significantly modulated these virulence-associated phenotypes. Specifically, gentamicin enhanced biofilm formation in ST1959, a finding consistent with reports that aminoglycosides can induce a bacterial stress response promoting biofilm development (Sato et al., 2018). This phenomenon raises the clinical concern that sublethal exposure during therapy might inadvertently foster bacterial persistence. In contrast, levofloxacin exhibited potent inhibitory effects, suppressing both biofilm formation and markedly reducing motility. This aligns with the known capacity of fluoroquinolones to interfere with key bacterial processes such as quorum sensing and pilus assembly, thereby curbing biofilm formation and motility (Aboelenin et al., 2024; Zhang et al., 2024; Baraka et al., 2025). Recent studies have revealed promising strategies to inhibit *A. baumannii* biofilm formation, a key contributor to multidrug resistance. Significant efficacy has been demonstrated by essential oils and natural compounds; for instance, cinnamaldehyde, onion ethanolic extract, Tween 80, and ginger essential oil achieved 70.8%–77.3% biofilm inhibition at low concentrations (Yadav et al., 2024). Synthetic approaches, such as *N*-acyl-2-aminopyrimidine derivatives, have also showed potent activity by disrupting quorum sensing and production of extracellular polysaccharide substances (Jia et al., 2022). Notably, conventional antibiotics like minocycline and polymyxin B were effective in preventing biofilm formation in >90% and 54% of isolates, respectively, at clinically achievable doses (Beganovic et al., 2019). Additionally, macrolides like erythromycin targeted the quorum sensing system (downregulating abaI and abaR genes), leading to biofilm disruption (Dong et al., 2024). Novel agents including cannabidiol (CBD), not only inhibit and eradicate biofilms but also synergiz with conventional antibiotics (e.g., colistin and meropenem), reducing effective concentrations by up to 1000-fold through membrane damage mechanisms (Yosboonruang et al., 2025). Our results suggest that, beyond direct bactericidal activity, levofloxacin may exert an “anti-virulence” effect at sub-MIC concentrations by impairing colonization and dissemination capabilities. This providing a novel rationale for its potential application in combination therapies or decolonization strategies.

## 5 Conclusion

This NICU outbreak was driven by a CRAB ST195 clone harboring a conserved and complex resistome. The isolates exhibiting the strongest biofilm formation coupled with the poorest motility, which were predominantly recovered from ETTs, likely represent a key adaptive strategy for successful persistence on medical devices. Our research reveals genotypic and phenotypic heterogeneity within a high-risk clone and delineate how subinhibitory antibiotic concentrations can critically modulate these virulence-associated phenotypes. These insights underscore the necessity of integrating both genetic background and phenotypic plasticity in infection control paradigms. The potent dual inhibitory effects of levofloxacin on biofilm formation and motility warrant further mechanistic and clinical exploration, potentially paving the way for novel therapeutic strategies against resilient CRAB infections.

## Data Availability

The raw data supporting the conclusions of this article will be made available by the authors, without undue reservation.

## References

[B1] Ababneh Q. Al Sbei S. Jaradat Z. Syaj S. Aldaken N. Ababneh H. . (2023). Extensively drug-resistant *Acinetobacter baumannii*: role of conjugative plasmids in transferring resistance. PeerJ 11, e14709. doi: 10.7717/peerj.14709, PMID: 36718445 PMC9884047

[B2] Aboelenin A. M. El-Mowafy M. Saleh N. M. Shaaban M. I. Barwa R. (2024). Ciprofloxacin- and levofloxacin-loaded nanoparticles efficiently suppressed fluoroquinolone resistance and biofilm formation in *Acinetobacter baumannii*. Sci. Rep. 14, 3125. doi: 10.1038/s41598-024-53441-1, PMID: 38326515 PMC10850473

[B3] Arciola C. R. Campoccia D. Montanaro L. (2018). Implant infections: adhesion, biofilm formation and immune evasion. Nat. Rev. Microbiol. 16, 397–409. doi: 10.1038/s41579-018-0019-y, PMID: 29720707

[B4] Armalyte J. Cepauskas A. Sakalyte G. Martinkus J. Skerniskyte J. Martens C. . (2023). A polyamine acetyltransferase regulates the motility and biofilm formation of *Acinetobacter baumannii*. Nat. Commun. 14, 3531. doi: 10.1038/s41467-023-39316-5, PMID: 37316480 PMC10267138

[B5] Ayoub Moubareck C. Hammoudi Halat D. (2020). Insights into Acinetobacter baumannii: A Review of Microbiological, Virulence, and Resistance Traits in a Threatening Nosocomial Pathogen. Antibiotics (Basel) 9, 119. doi: 10.3390/antibiotics9030119, PMID: 32178356 PMC7148516

[B6] Baraka K. Abozahra R. Khalaf E. Bennaya M. E. Abdelhamid S. M. (2025). Repurposing of paroxetine and fluoxetine for their antibacterial effects against clinical *Pseudomonas aeruginosa* isolates in Egypt. AIMS Microbiol. 11, 126–149. doi: 10.3934/microbiol.2025007, PMID: 40161243 PMC11950684

[B7] Beganovic M. Luther M. K. Daffinee K. E. LaPlante K. L. (2019). Biofilm prevention concentrations (BPC) of minocycline compared to polymyxin B, meropenem, and amikacin against *Acinetobacter baumannii*. Diagn. Microbiol. Infect. Dis. 94, 223–226. doi: 10.1016/j.diagmicrobio.2019.01.016, PMID: 30827805

[B8] Clemmer K. M. Bonomo R. A. Rather P. N. (2011). Genetic analysis of surface motility in *Acinetobacter baumannii*. Microbiol. (Reading) 157, 2534–2544. doi: 10.1099/mic.0.049791-0, PMID: 21700662 PMC3352170

[B9] Deshmukh-Reeves E. Shaw M. Bilsby C. Gourlay C. W. (2025). Biofilm formation on endotracheal and tracheostomy tubing: A systematic review and meta-analysis of culture data and sampling method. Microbiologyopen 14, e70032. doi: 10.1002/mbo3.70032, PMID: 40619937 PMC12230368

[B10] Dong H. Sun J. Liu Y. Li Q. Huang J. Xu P. . (2024). Erythromycin disrupts *Acinetobacter baumannii* biofilms through destruction of the quorum sensing system. Med. (Baltimore) 103, e38341. doi: 10.1097/md.0000000000038341, PMID: 39252274 PMC11383728

[B11] Hu Z. Wang Z. Liu D. Chen P. Wang H. Chen Y. . (2011). Clinical and molecular microbiological characteristics of carbapenem-resistant *Acinetobacter baumannii* strains in an NICU. Pediatr. Int. 53, 867–872. doi: 10.1111/j.1442-200X.2011.03397.x, PMID: 21605279

[B12] Iovleva A. Fowler V. G. Jr. Doi Y. (2025). Treatment approaches for carbapenem-resistant *Acinetobacter baumannii* infections. Drugs 85, 21–40. doi: 10.1007/s40265-024-02104-6, PMID: 39607595 PMC11950131

[B13] Isler B. Doi Y. Bonomo R. A. Paterson D. L. (2019). New Treatment Options against Carbapenem-Resistant *Acinetobacter baumannii* Infections. Antimicrob. Agents Chemother. 63, 01110-18. doi: 10.1128/aac.01110-18, PMID: 30323035 PMC6325237

[B14] Jain M. Sharma A. Sen M. K. Rani V. Gaind R. Suri J. C. (2019). Phenotypic and molecular characterization of *Acinetobacter baumannii* isolates causing lower respiratory infections among ICU patients. Microb. Pathog. 128, 75–81. doi: 10.1016/j.micpath.2018.12.023, PMID: 30562602

[B15] Jia X. M. Cheng C. Liu T. Zhao Y. L. Guo B. Tang L. . (2022). Synthesis and antibiofilm evaluation of N-acyl-2-aminopyrimidine derivatives against *Acinetobacter baumannii*. Bioorg Med. Chem. 76, 117095. doi: 10.1016/j.bmc.2022.117095, PMID: 36442439

[B16] Kamolvit W. Sidjabat H. E. Paterson D. L. (2015). Molecular epidemiology and mechanisms of carbapenem resistance of *Acinetobacter* spp. in Asia and Oceania. Microb. Drug Resist. 21, 424–434. doi: 10.1089/mdr.2014.0234, PMID: 25714653

[B17] Kumar S. Jan R. A. Fomda B. A. Rasool R. Koul P. Shah S. . (2018). Healthcare-associated pneumonia and hospital-acquired pneumonia: bacterial aetiology, antibiotic resistance and treatment outcomes: A study from north India. Lung 196, 469–479. doi: 10.1007/s00408-018-0117-7, PMID: 29691645

[B18] Lee C. R. Lee J. H. Park M. Park K. S. Bae I. K. Kim Y. B. . (2017). Biology of *Acinetobacter baumannii:* pathogenesis, antibiotic resistance mechanisms, and prospective treatment options. Front. Cell Infect. Microbiol. 7. doi: 10.3389/fcimb.2017.00055, PMID: 28348979 PMC5346588

[B19] Liu L. L. Ji S. J. Ruan Z. Fu Y. Fu Y. Q. Wang Y. F. . (2015). Dissemination of blaOXA-23 in Acinetobacter spp. in China: main roles of conjugative plasmid pAZJ221 and transposon Tn2009. Antimicrob. Agents Chemother. 59, 1998–2005. doi: 10.1128/AAC.04574-14, PMID: 25605357 PMC4356780

[B20] Luo Q. Chang M. Lu P. Guo Q. Jiang X. Xiao T. . (2025). Genomic epidemiology and phylodynamics of *Acinetobacter baumannii* bloodstream isolates in China. Nat. Commun. 16, 3536. doi: 10.1038/s41467-025-58772-9, PMID: 40229304 PMC11997098

[B21] Luo Q. Lu P. Chen Y. Shen P. Zheng B. Ji J. . (2024). ESKAPE in China: epidemiology and characteristics of antibiotic resistance. Emerg. Microbes Infect. 13, 2317915. doi: 10.1080/22221751.2024.2317915, PMID: 38356197 PMC10896150

[B22] Mohd Sazlly Lim S. Zainal Abidin A. Liew S. M. Roberts J. A. Sime F. B. (2019). The global prevalence of multidrug-resistance among *Acinetobacter baumannii* causing hospital-acquired and ventilator-associated pneumonia and its associated mortality: A systematic review and meta-analysis. J. Infect. 79, 593–600. doi: 10.1016/j.jinf.2019.09.012, PMID: 31580871

[B23] Mugnier P. D. Poirel L. Naas T. Nordmann P. (2010). Worldwide dissemination of the blaOXA-23 carbapenemase gene of *Acinetobacter baumannii*. Emerg. Infect. Dis. 16, 35–40. doi: 10.3201/eid1601.090852, PMID: 20031040 PMC2874364

[B24] Muller C. Reuter S. Wille J. Xanthopoulou K. Stefanik D. Grundmann H. . (2023). A global view on carbapenem-resistant Acinetobacter baumannii. mBio 14, e0226023. doi: 10.1128/mbio.02260-23, PMID: 37882512 PMC10746149

[B25] O'Toole G. A. Kolter R. (1998). Initiation of biofilm formation in Pseudomonas fluorescens WCS365 proceeds via multiple, convergent signalling pathways: a genetic analysis. Mol. Microbiol. 28, 449–461. doi: 10.1046/j.1365-2958.1998.00797.x, PMID: 9632250

[B26] Pannek S. Higgins P. G. Steinke P. Jonas D. Akova M. Bohnert J. A. . (2006). Multidrug efflux inhibition in *Acinetobacter baumannii*: comparison between 1-(1-naphthylmethyl)-piperazine and phenyl-arginine-beta-naphthylamide. J. Antimicrob. Chemother. 57, 970–974. doi: 10.1093/jac/dkl081, PMID: 16531429

[B27] Penesyan A. Paulsen I. T. Kjelleberg S. Gillings M. R. (2021). Three faces of biofilms: a microbial lifestyle, a nascent multicellular organism, and an incubator for diversity. NPJ Biofilms Microbiomes 7, 80. doi: 10.1038/s41522-021-00251-2, PMID: 34759294 PMC8581019

[B28] Percival S. L. Suleman L. Vuotto C. Donelli G. (2015). Healthcare-associated infections, medical devices and biofilms: risk, tolerance and control. J. Med. Microbiol. 64, 323–334. doi: 10.1099/jmm.0.000032, PMID: 25670813

[B29] Phatigomet M. Thatrimontrichai A. Maneenil G. Dissaneevate S. Janjindamai W. (2022). Risk factors for 30-day mortality in neonates with carbapenem-resistant *A. baumannii* sepsis. Pediatr. Infect. Dis. J. 41, 1012–1016. doi: 10.1097/INF.0000000000003721, PMID: 36375101

[B30] Poirel L. Nordmann P. (2006). Carbapenem resistance in *Acinetobacter baumannii:* mechanisms and epidemiology. Clin. Microbiol. Infect. 12, 826–836. doi: 10.1111/j.1469-0691.2006.01456.x, PMID: 16882287

[B31] Rizk M. A. Abou El-Khier N. T. (2019). Aminoglycoside resistance genes in Acinetobacter baumannii clinical isolates. Clin. Lab. 65. doi: 10.7754/Clin.Lab.2019.190103, PMID: 31307186

[B32] Royer S. de Campos P. A. Araujo B. F. Ferreira M. L. Goncalves I. R. Batistao D. . (2018). Molecular characterization and clonal dynamics of nosocomial blaOXA-23 producing XDR *Acinetobacter baumannii*. PloS One 13, e0198643. doi: 10.1371/journal.pone.0198643, PMID: 29889876 PMC5995351

[B33] Sato Y. Unno Y. Ubagai T. Ono Y. (2018). Sub-minimum inhibitory concentrations of colistin and polymyxin B promote *Acinetobacter baumannii* biofilm formation. PloS One 13, e0194556. doi: 10.1371/journal.pone.0194556, PMID: 29554105 PMC5858813

[B34] Sharma S. Kaushik V. Kulshrestha M. Tiwari V. (2023). Different efflux pump systems in Acinetobacter baumannii and their role in multidrug resistance. Adv. Exp. Med. Biol. 1370, 155–168. doi: 10.1007/5584_2023_771, PMID: 36971967

[B35] Sung J. Y. Koo S. H. Kim S. Kwon G. C. (2016). Persistence of Multidrug-Resistant *Acinetobacter baumannii* Isolates Harboring blaOXA-23 and bap for 5 Years. J. Microbiol. Biotechnol. 26, 1481–1489. doi: 10.4014/jmb.1604.04049, PMID: 27221112

[B36] Tacconelli E. Carrara E. Savoldi A. Harbarth S. Mendelson M. Monnet D. L. . (2018). Discovery, research, and development of new antibiotics: the WHO priority list of antibiotic-resistant bacteria and tuberculosis. Lancet Infect. Dis. 18, 318–327. doi: 10.1016/s1473-3099(17)30753-3, PMID: 29276051

[B37] Tsiatsiou O. Iosifidis E. Katragkou A. Dimou V. Sarafidis K. Karampatakis T. . (2015). Successful management of an outbreak due to carbapenem-resistant *Acinetobacter baumannii* in a neonatal intensive care unit. Eur. J. Pediatr. 174, 65–74. doi: 10.1007/s00431-014-2365-8, PMID: 24985124

[B38] Ulu-Kilic A. Gundogdu A. Cevahir F. Kilic H. Gunes T. Alp E. (2018). An outbreak of bloodstream infection due to extensively resistant *Acinetobacter baumannii* among neonates. Am. J. Infect. Control 46, 154–158. doi: 10.1016/j.ajic.2017.08.007, PMID: 28958447

[B39] Vogiantzi G. Metallinou D. Tigka M. Deltsidou A. Nanou C. I. (2024). Bloodstream infections in the neonatal intensive care unit: A systematic review of the literature. Cureus 16, e68057. doi: 10.7759/cureus.68057, PMID: 39347186 PMC11438544

[B40] Wang M. Ge L. Chen L. Komarow L. Hanson B. Reyes J. . (2024). Clinical outcomes and bacterial characteristics of Carbapenem-resistant *Acinetobacter baumannii* among patients from different global regions. Clin. Infect. Dis. 78, 248–258. doi: 10.1093/cid/ciad556, PMID: 37738153 PMC10874260

[B41] Wei H. M. Hsu Y. L. Lin H. C. Hsieh T. H. Yen T. Y. Lin H. C. . (2015). Multidrug-resistant *Acinetobacter baumannii* infection among neonates in a neonatal intensive care unit at a medical center in central Taiwan. J. Microbiol. Immunol. Infect. 48, 531–539. doi: 10.1016/j.jmii.2014.08.025, PMID: 25442873

[B42] Yadav P. Shrestha S. Basyal D. Tiwari A. Sah R. Sah A. K. . (2024). Characterization and biofilm inhibition of multidrug-resistant *Acinetobacter baumannii* isolates. Int. J. Microbiol. 2024, 5749982. doi: 10.1155/ijm/5749982, PMID: 39758150 PMC11699987

[B43] Yosboonruang A. Kiddee A. Siriphap A. Pook-In G. Suwancharoen C. Duangjai A. . (2025). Potential of Cannabidiol (CBD) to overcome extensively drug-resistant *Acinetobacter baumannii*. BMC Complement Med. Ther. 25, 308. doi: 10.1186/s12906-025-05056-w, PMID: 40817249 PMC12357442

[B44] Zarrilli R. Di Popolo A. Bagattini M. Giannouli M. Martino D. Barchitta M. . (2012). Clonal spread and patient risk factors for acquisition of extensively drug-resistant *Acinetobacter baumannii* in a neonatal intensive care unit in Italy. J. Hosp Infect. 82, 260–265. doi: 10.1016/j.jhin.2012.08.018, PMID: 23102814

[B45] Zhang Y. Jiao F. Zeng D. Yu X. Zhou Y. Xue J. . (2024). Synergistic Effects of Pyrrosia lingua Caffeoylquinic Acid Compounds with Levofloxacin Against Uropathogenic *Escherichia coli*: Insights from Molecular Dynamics Simulations, Antibiofilm, and Antimicrobial Assessments. Molecules. 29, 5679. doi: 10.3390/molecules29235679, PMID: 39683837 PMC11643949

